# Plerixafor and resatorvid inhibit hepatitis B virus *in vitro* by upregulating elongation factor Tu GTP-binding domain containing 2

**DOI:** 10.3389/fcimb.2023.1118801

**Published:** 2023-02-20

**Authors:** Jinyuan Cai, Yuwen Li, Pingping Hu, Ruirui Xu, Hui Yuan, Wen Zhang, Tiantong Feng, Rui Liu, Wenting Li, Chuanlong Zhu

**Affiliations:** ^1^ 1Department of Infectious Disease, The First Affiliated Hospital of Nanjing Medical University, Nanjing, China; ^2^ Department of Infectious Disease, Zhongda Hospital, Southeast University, Nanjing, China; ^3^ Department of Pediatrics, The First Affiliated Hospital of Nanjing Medical University, Nanjing, China; ^4^ Department of Infectious and Tropical Diseases, The Second Affiliated Hospital of Hainan Medical University, Hainan, China

**Keywords:** hepatitis B virus, small-molecule agents, plerixafor, resatorvid, EFTUD2

## Abstract

**Background:**

An increase in the demand for a functional cure has accelerated research on new methods of therapy for chronic hepatitis B, which is mainly focused on restoring antiviral immunity for controlling viral infections. Previously, we had described elongation factor Tu GTP-binding domain containing 2 (EFTUD2) as an innate immune regulator and suggested that it might be an antiviral target.

**Methods:**

In this study, we generated the Epro-LUC-HepG2 cell model for screening compounds that target EFTUD2. Plerixafor and resatorvid were screened from 261 immunity and inflammation-related compounds due to their ability to highly upregulate EFTUD2. The effects of plerixafor and resatorvid on hepatitis B virus (HBV) were examined in HepAD38 cells and HBV-infected HepG2-NTCP cells.

**Results:**

The dual-luciferase reporter assays showed that the EFTUD2 promoter hEFTUD2pro-0.5 kb had the strongest activity. In Epro-LUC-HepG2 cells, plerixafor and resatorvid significantly upregulated the activity of the EFTUD2 promoter and the expression of the gene and protein. In HepAD38 cells and HBV-infected HepG2-NTCP cells, treatment with plerixafor and resatorvid strongly inhibited HBsAg, HBV DNA, HBV RNAs, and cccDNA in a dose-dependent manner. Furthermore, the anti-HBV effect was enhanced when entecavir was administered along with either of the previous two compounds, and the effect could be blocked by knocking down EFTUD2.

**Conclusion:**

We established a convenient model for screening compounds that target EFTUD2 and further identified plerixafor and resatorvid as novel HBV inhibitors *in vitro*. Our findings provided information on the development of a new class of anti-HBV agents that act on host factors rather than viral enzymes.

## Introduction

Chronic hepatitis B (CHB) is a major risk factor for liver cirrhosis and hepatocellular carcinoma (Xia and Liang, 2019). Interferon (IFN) can effectively perform virological clearance in chronic hepatitis B virus (HBV) infection, but its clearance rate is low and only a few patients benefit from IFN-based therapy (Geng et al., 2018). Additionally, although nucleoside analogs (NAs) can inhibit the replication of HBV DNA, they are inefficient in removing the hepatitis B surface antigen (HBsAg). Therefore, efficacious anti-HBV therapeutic methods need to be developed (Levrero et al., 2018).

The innate immunity of the host is the first line of defense against the invasion of viruses and determines the outcome of infection (Horner and Gale, 2013). In another study, we identified the elongation factor Tu GTP-binding domain-containing 2 (EFTUD2) as a novel host factor that can counter hepatitis C virus (HCV) infection (Zhu et al., 2015). EFTUD2 encodes a GTPase responsible for pre-mRNA splicing (Janssen et al., 2005) and can regulate the innate immune response by alternatively splicing the mRNA of myeloid differentiation factor 88 (MyD88) (De Arras et al., 2014), which is a key factor involved in type I IFN response and many viral infections (Saikh, 2021). HBsAg and HBeAg can suppress the binding of MyD88 and decrease IFN signaling (De Arras et al., 2014). Single-nucleotide polymorphism analysis showed that EFTUD2 rs3809756 polymorphism is significantly associated with susceptibility to HBV infection (Tian et al., 2022). However, the effect of EFTUD2 on HBV replication is not known. In this study, we found that HBV is inhibited by EFTUD2 in different cell models, and then, proposed an approach for screening new anti-HBV agents to provide more options for the immunotherapy of CHB patients with poor response to IFN therapy.

We screened 261 compounds associated with immunity and inflammation and found that plerixafor and resatorvid could significantly upregulate the expression of EFTUD2 and reduce HBV replication *in vitro*. Moreover, plerixafor and resatorvid inhibited the transcriptional activity of cccDNA to suppress HBV RNA synthesis and HBsAg secretion and showed anti-HBV activity. These findings provided insights into the pharmacodynamics of plerixafor and resatorvid. Additionally, their antiviral effects increased when combined with entecavir (ETV). Thus, plerixafor and resatorvid may be considered as valuable candidates for the treatment of HBV in the future.

## Materials and methods

### Chemical compounds

The 261 screened compounds associated with immunity and inflammation were purchased from MedChemExpress (MCE, HY-L007) and stored at –80°C at the concentration recommended by the manufacturer. They were diluted in a medium to the desired concentration before use.

### Cell culture

The HepG2 cells were purchased from the Cell Bank of Type Culture Collection of the Chinese Academy of Sciences. The HepAD38 and HepG2-NTCP cells were gifts from the Institute of Blood Transfusion, Chinese Academy of Medical Sciences and Peking Union Medical College, Chengdu, Sichuan Province, China. All cells were cultured in Dulbecco’s Modified Eagle medium (DMEM, Gibco) containing 10% fetal bovine serum (FBS, Gibco) and incubated in a humidified atmosphere containing 5% CO_2_ at 37°C. To maintain the stably transfected HBV genome, the HepAD38 cells were grown with 1 µg/mL doxycycline (MCE) and 400 µg/mL G418 (Thermo Fisher Scientific).

### Virus extraction and infection

The supernatants of the HepaAD38 cells were concentrated 100-fold by ultracentrifugation as HBV inoculums. The HBV stock titer (genome equivalents [GEq] per milliliter) was measured by performing qPCR.

The HepG2-NTCP cells were incubated with 1,000 GEq/cell of HBV in a medium containing 4% (w/v) polyethylene glycol 8000 (PEG 8000) for 16 h. After discarding the viral mixture, the cells were rinsed thrice with PBS, and cultured in a fresh medium containing different agents at various concentrations. The medium was replaced every 2 days.

### Detection of HBsAg

Briefly, different concentrations of agents were added to the plates 24 h after seeding the cells. Then, the supernatants were collected at predetermined time points and the secretion of HBsAg was detected using enzyme-linked immunosorbent assay (ELISA) kits (KHB, Shanghai, China) following the manufacturer’s instructions. The absorbance was measured at 450 nm. The supernatant from the HepAD38 cells was diluted 20 times and the supernatant from the other cells was used as primary samples. All OD values were between 0.5 and 3.5.

### Plasmid and siRNA

The small interfering RNA (siRNA) targeting EFTUD2 and the plasmid pEFTUD2 were purchased from GenePharma (Shanghai, China). They were transfected into HepAD38 cells and HBV-infected HepG2-NTCP cells with the Lipo3000 reagent (Thermo Fisher Scientific, USA) following the manufacturer’s instructions. The knockout efficiency was determined by performing RT-qPCR for detecting the EFTUD2 mRNA and a Western blotting assay was performed for detecting the EFTUD2 protein.

### Cell cytotoxicity assay

The effect of the compounds on cell cytotoxicity was measured by performing the MTT [3-(4,5-dimethylthiazol-2-yl)-2,5-diphenyltetrazolium bromide] assay (Sigma-Aldrich). The cells (2 × 10^3^/well) were seeded into 96-well plates in 100 µL of DMEM and cultured at 37°C for 24 h. Then, the culture medium was replaced with a fresh medium containing various concentrations of compounds for a certain number of days. After the cells were cultured for a specific period, 10 µL of MTT (5 mg/mL) was added to each well. After incubation for 4 h, the supernatant was removed and the cells were lysed in 100 µL of DMSO (Solarbio). Then, the cytotoxicity was determined by analyzing MTT absorbance at 490 nm.

### Luciferase reporter assay

The HepG2 cells were seeded in 12-well-plates at a concentration of 2 × 10^5^ cells/well for 24 h, and then, transfected with a promoter-reporter plasmid plus vectors containing the gene of interest by Lipofectamine™ 3000 (Invitrogen). The Renilla luciferase reporter plasmid was used as the internal control of transfection efficiency. After transfection for 48 h, the luciferase activity was measured using a GloMax microplate luminometer (Promega).

All vectors used in this study were purchased from GenePharma (Shanghai, China). The restriction enzymes, different modification enzymes, and T4-DNA ligase were purchased from MBI Fermentas (Ontario, Canada).

### Hirt extraction of cccDNA and analysis

To selectively extract HBV cccDNA, infected HepG2-NTCP cells were lysed in 6-cm dishes with 1 mL of lysis buffer at 37°C for 60 min, and then, incubated with 0.25 mL of 2.5 M KCl overnight at 4°C. The lysis buffer contained 50 mM Tris–HCl (pH 7.4), 10 mM EDTA, 150 mM NaCl, and 1% SDS, without proteinase K. The lysate was clarified by centrifugation at 12,000 g for 30 min at 4°C. Viral DNA was extracted with phenol and phenol: chloroform, precipitated in an equal volume of isopropanol containing 20 µg glycogen (Roche), and dissolved in TE buffer. The prepared DNA sample was then treated with plasmid-safe adenosine triphosphate (ATP)-dependent deoxyribonuclease DNase (Epicentre Technologies) following the manufacturer’s instructions.

The treated Hirt DNA was subjected to Taq-man probe RT-qPCR for detecting the HBV cccDNA levels; the specific primers and the probe used are listed in the Supplementary (**Table. S1**).

### Real-time PCR

HBV DNA was extracted using the QIAamp DNA Mini kit (Qiagen, Germany) and total RNA was extracted using TRIzol reagent (Invitrogen) following the manufacturer’s instructions. The DNA and RNA samples were quantified by Nanodrop 2000 (Thermo scientific). The cDNA was synthesized from about 1,000 ng of RNA using the PrimeScript RT kit (Takara).

The levels of HBV genomic DNA, HBV RNAs, and EFTUD2 mRNA were detected by real-time PCR analysis, using SYBR Green (Roche, Germany) in the Applied Biosystems QuantStudio 3 Real-Time PCR System. The expression of the target genes was normalized by glyceraldehyde 3-phosphate dehydrogenase (GAPDH). The primers used are detailed in the Supplementary (**Table. S1**).

### Western blot

The proteins were separated by performing SDS polyacrylamide gel electrophoresis. Then, they were transferred onto polyvinylidene difluoride (PVDF; Thermo Scientific) membranes, blocked with 5% gelatin in TBST, and incubated with the primary antibodies at 4°C overnight. After washing the membranes thrice, the secondary antibodies (1:8000) conjugated to horseradish peroxidase (HRP) were added, and the mixture was incubated for 1 h at room temperature. The images were recorded using the enhanced chemiluminescence (ECL) system (Invitrogen). The mouse anti-GAPDH and the rabbit anti-EFTUD2 were purchased from Abcam. The HRP-conjugated enhanced ECL goat anti-rabbit immunoglobulin G (IgG) and HRP-conjugated ECL goat anti-mouse IgG were purchased from Bioss (Beijing, China).

### Southern blot

The DNA samples were separated on a 0.9% agarose gel and transferred onto a nylon membrane overnight (Roche, Germany). After UV cross-linking and prehybridization, hybridization was performed by rotating and incubating the membrane with digoxigenin-labeled HBV-specific DNA probes, using a random primed DNA labeling kit (Roche, Germany). The radioactive signals were detected using the GelDocXR System (Bio-Rad).

### Statistical analysis

Statistical analyses were performed using GraphPad Prism (GraphPad version 7). All experiments were repeated at least thrice. The data were presented as the mean ± standard deviation (SD) and comparisons were made by performing unpaired Student’s t-tests. All differences were considered to be statistically significant at p < 0.05.

## Results

### EFTUD2 has an anti-HBV effect *in vitro*

**Figure 1:**
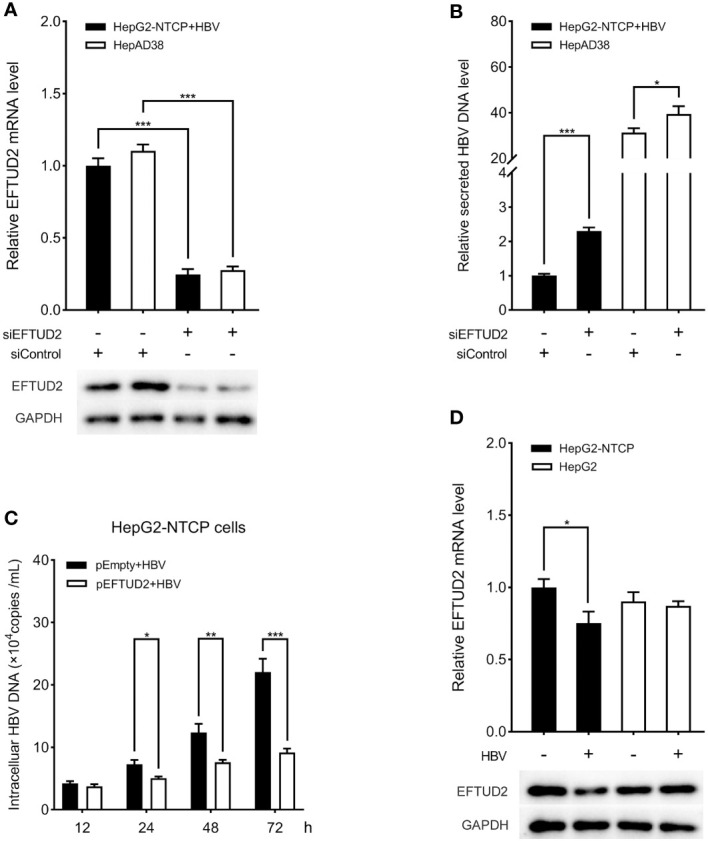
EFTUD2 had an anti-HBV effect *in vitro*. **(A)** The efficacy of the knockdown of EFTUD2 was determined by qPCR and Western blot analyses in HepAD38 cells and HepG2-NTCP cells. **(B)** The knockdown of EFTUD2 improved the HBV DNA levels. **(C)** EFTUD2 overexpression inhibited HBV replication at 12, 24, 48, 72 h postinfection. **(D)** Real-time PCR and Western blot analyses showed that EFTUD2 was downregulated in HepG2-NTCP and HepG2 cells treated with HBV particles at 48 h. *, *P* < 0.05; **, *P* < 0.01; ***, *P* < 0.001.

To elucidate the role of EFTUD2 in HBV infection, we first silenced EFTUD2 in HepAD38 and HepG2-NTCP cells. Real-time PCR and western blotting assays showed efficient knockdown of EFTUD2 (>70%, *P* < 0.001) in both cell lines (**Figure 1A**). We found that knocking down EFTUD2 significantly enhanced HBV replication, based on the results of real-time PCR analysis (**Figure 1B**). In contrast, overexpression of EFTUD2 resulted in decreased HBV DNA levels at 24, 48 and 72 h postinfection (**Figure 1C**), indicating its role in restricting HBV infection at the viral postentry stage. Moreover, EFTUD2 expression decreased in HepG2-NTCP cells at 48 h postinfection (*P* < 0.05) (**Figure 1D**), which may be caused by immune escape after HBV entry.

### Analysis of the activity of the EFTUD2 promoters and construction of the Epro-LUC-HepG2 cell line

**Figure 2:**
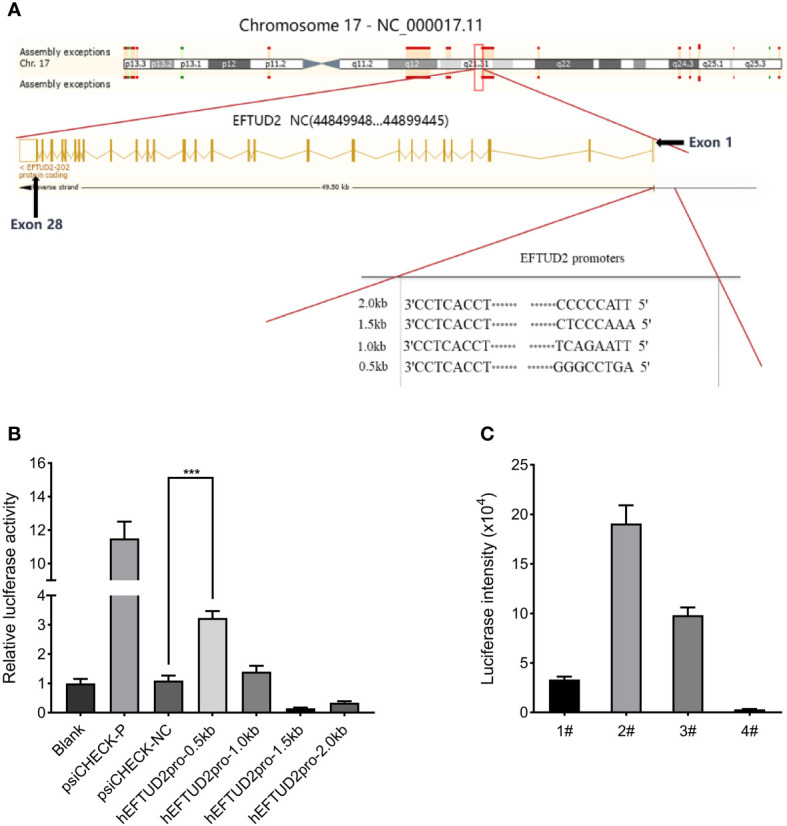
Construction of the Epro-LUC-HepG2 cell line. **(A)** Prediction of the EFTUD2 promoter sequences was performed according to the ENCODE data. **(B)** The luciferase analysis showed that the hEFTUD2pro-0.5 kb promoter had the strongest activity. **(C)** Stable transgenic Epro-LUC-HepG2 cell line (2#) was selected and the luciferase intensity was displayed. ***, *P* < 0.001.

According to the ENCODE data, we predicted a promoter region near the exon1 of EFTUD2, where DNA endonuclease hypersensitive site (DHS) and transcriptional activity enhancement marker H3K27Ac are enriched (**Figure 2A**). Four fragments of 0.5 kbp, 1 kbp, 1.5 kbp, and 2 kbp from –1 to –2 kbp upstream of the transcriptional initiation site were selected. The PCR products were digested with the Nhel and BglII enzymes and cloned into the psiCHECK-2 vector to generate the recombinant plasmid of the promoter-luciferase reporter gene. All constructs were checked for the correct size by agarose gel electrophoresis (**Figure S1A**), verified by DNA sequencing, and transfected into HepG2 cells for 48 h. The results of the luciferase analysis suggested that the hEFTUD2pro-0.5 kb promoter had the strongest activity (3.2-fold, *P* < 0.05) (**Figure 2B**).

Therefore, the hEFTUD2pro-0.5 kb promoter sequence was fused to the firefly luciferase reporter gene, digested with Nhel and BamHI enzymes, and then, inserted into the LV6 vector to construct the LV6-Epro0.5-LUC luciferase reporter plasmid (**Figure S1B**). After transfection with the plasmid *via* lentivirus and selection with puromycin, the HepG2 transfected cell line was processed by monoclonal screening and the Epro-LUC-HepG2 cell line (2#) was obtained (**Figure 2C**).

### Identification of plerixafor and resatorvid as upregulators of EFTUD2

**Figure 3:**
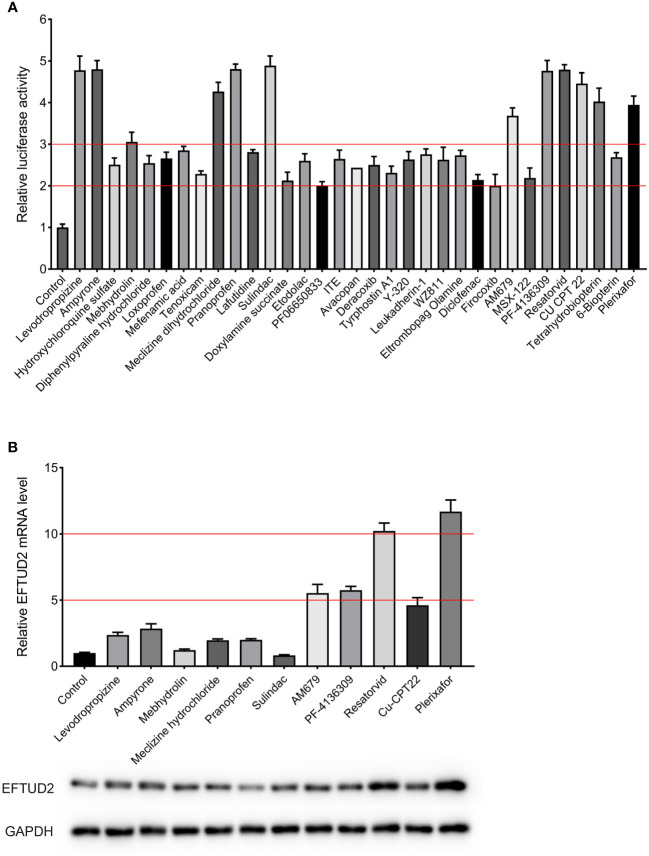
Screening compounds that upregulated the EFTUD2 promoter activity, genes, and proteins. **(A)** The luciferase analysis showed that 33 compounds increased the activity of the EFTUD2 promoter by more than two times, and 12 compounds increased the activity by more than three times. **(B)** Real-time PCR and Western blot analyses showed that plerixafor and resatorvid had the strongest ability to upregulate the activity of the EFTUD2 promoter among the 11 compounds (tetrahydrobiopterin was abandoned due to its obvious cytotoxicity).

The established Epro-LUC-HepG2 cell line was used for screening the 261 compounds from the HY-L007 compound library provided by MCE. The cytotoxicity of these compounds was evaluated by performing the MTT assay (partly shown in **Figure S2**). During the screening process, 0.1% DMSO was used as the negative control in each experimental setup. We found that 33 compounds increased luciferase activity by more than 2 times, 11 of which were selected as the candidates for further experiments and increased luciferase activity by more than 3 times (**Figure 3A**). To verify that they upregulate the expression of EFTUD2, Epro-LUC-HepG2 cells were treated with these compounds for 3 days and the expression of the EFTUD2 mRNA and protein was quantified. Among the 11 candidates, plerixafor and resatorvid increased the EFTUD2 mRNA level by more than 10 times and increased the EFTUD2 protein level by more than two times. Thus, plerixafor and resatorvid were selected in this study due to their low cytotoxicity and ability to significantly upregulate the EFTUD2 promoter activity, along with its mRNA and protein levels (**Figure 3B**). The characteristics of the two agents and their effects on the viability of HepAD38 and HepG2-NTCP cells were shown in **Figure S3**.

### Inhibitory effects of plerixafor and resatorvid on HBV replication

**Figure 4:**
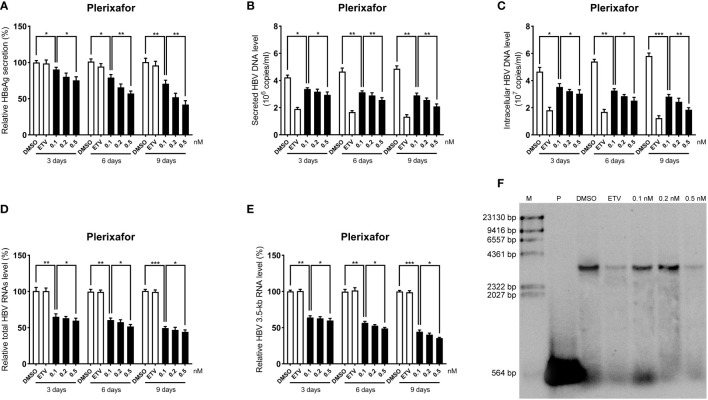
Plerixafor inhibited HBV replication in HepAD38 cells. HepAD38 cells were treated with 0.1% DMSO, 0.1, 0.2, or 0.5 nM plerixafor and 25 nM ETV for 3, 6 and 9 days. The levels of HBsAg, secreted and intracellular HBV DNA, and HBV RNAs were detected. **(A)** The results of the ELISA showed a decrease in the level of HBsAg in the supernatant. **(B-E)** Real-time PCR analysis showed that plerixafor inhibited HBV DNA both in the supernatant and cells, as well as, HBV total RNAs and 3.5-kb RNA. **(F)** A Southern blot analysis was performed to determine the level of HBV DNA after 9 days of treatment. *, *P* < 0.05; **, *P* < 0.01; ***, *P* < 0.001.

**Figure 5:**
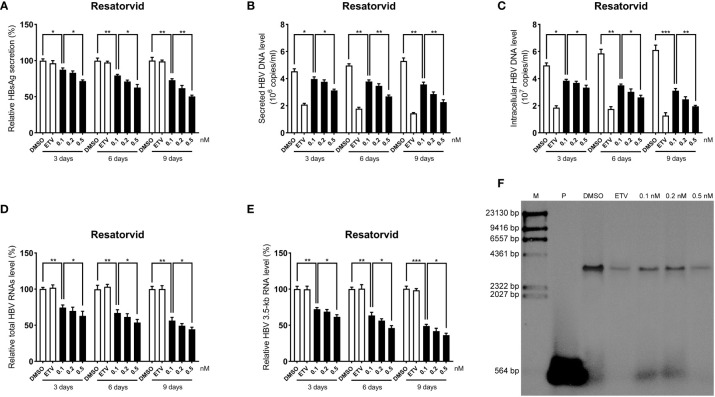
Resatorvid inhibited HBV replication in HepAD38 cells. HepAD38 cells were treated with 0.1% DMSO, 0.1, 0.2, or 0.5 nM resatorvid and 25 nM ETV for 3, 6 and 9 days. The levels of HBsAg, secreted and intracellular HBV DNA, and HBV RNAs were detected. **(A)** The results of the ELISA showed a decrease in the level of HBsAg in the supernatant. **(B-E)** Real-time PCR analysis showed that resatorvid inhibited HBV DNA both in the supernatant and cells, as well as, HBV total RNAs and 3.5-kb RNA. **(F)** A Southern blot analysis was performed to determine the level of HBV DNA after 9 days of treatment. *, *P* < 0.05; **, *P* < 0.01; ***, *P* < 0.001.

To further determine the anti-HBV activity of plerixafor and resatorvid, the HBV markers, including HBV RNAs, DNA, and HBsAg, were assessed in HepAD38 cells after treatment for 3, 6, and 9 days with the agent, respectively. The results of the ELISA showed that plerixafor significantly reduced the level of HBsAg in the supernatant. Specifically, after administering 0.1 nM plerixafor, the level of HBsAg decreased on the third (90%, *P* < 0.05), sixth (81%, *P* < 0.05), and ninth (68%, *P* < 0.01) day, compared to the level of HBsAg after DMSO treatment (**Figure 4A**). Similar results were found after administering 0.2 nM and 0.5 nM plerixafor (**Figure 4A**). Furthermore, treatment with 0.1 nM resatorvid reduced the level of HBsAg on the third (87%, *P* < 0.05), sixth (79%, *P* < 0.01), and ninth (73%, *P* < 0.01) day, compared to the level of HBsAg after DMSO treatment (**Figure 5A**). Similar results were found after treatment with 0.2 nM and 0.5 nM resatorvid (**Figure 5A**). Real-time PCR analysis showed that the secreted HBV DNA level and intracellular HBV DNA level also decreased significantly after treatment with plerixafor in a dose-dependent manner (**Figures 4B, C**, **5B, C**). To further validate our findings, Southern blot analysis was performed. The bands were approximately 3.2 kbp (**Figures 4F**, **5F**), which was similar to the full-length HBV genome; thus, indicating that intracellular HBV DNA was correctly detected. The HBV DNA in the cells decreased significantly after treatment for 10 days, indicating that plerixafor and resatorvid inhibited the replication of HBV DNA.

HBV cccDNA serves as the template for transcription of all four viral mRNAs (3.5, 2.4, 2.1, and 0.7 kb) (Ren et al., 2019). To determine whether the reduction of HBV DNA was due to a decrease in the mRNA levels, HBV RNAs were analyzed by real-time PCR analysis. The results showed that plerixafor and resatorvid significantly decreased the levels of total HBV RNAs and 3.5-kb RNA in a dose-dependent manner, whereas, ETV had no such effects (**Figures 4D, E**, **5D, E**). Together, these results suggested that plerixafor and resatorvid inhibited HBV replication in HepAD38 cells.

### Inhibitory effect of plerixafor and resatorvid on HBV replication in the HBV infection model

**Figure 6:**
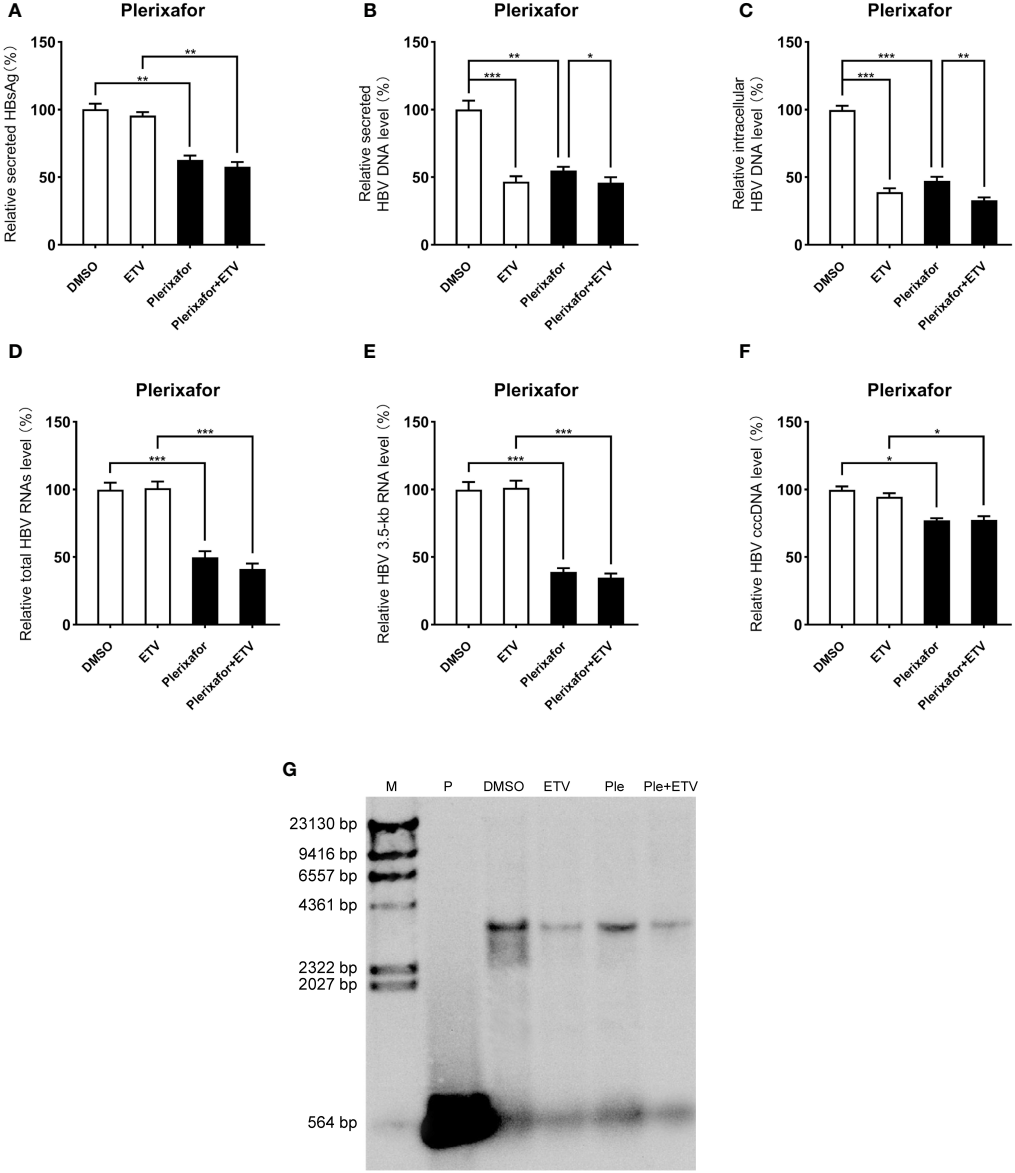
Plerixafor showed anti-HBV activity in the HBV infection model. HepG2-NTCP cells were infected with HBV particles (1,000 GEq/cell). Then, the HBV-infected cells were treated with 0.1% DMSO, 0.5 nM plerixafor or/and 25 nM ETV for 10 days. **(A)** The results of the ELISA showed that plerixafor decreased the HBsAg level in the supernatant. **(B-E)** Plerixafor significantly inhibited secreted and intracellular HBV DNA, total HBV RNAs, and the 3.5-kb RNA. **(F)** Plerixafor partly decreased the level of cccDNA. **(G)** A Southern blot analysis was performed to determine the reduction of HBV DNA. *, *P* < 0.05; **, *P* < 0.01; ***, *P* < 0.001.

**Figure 7:**
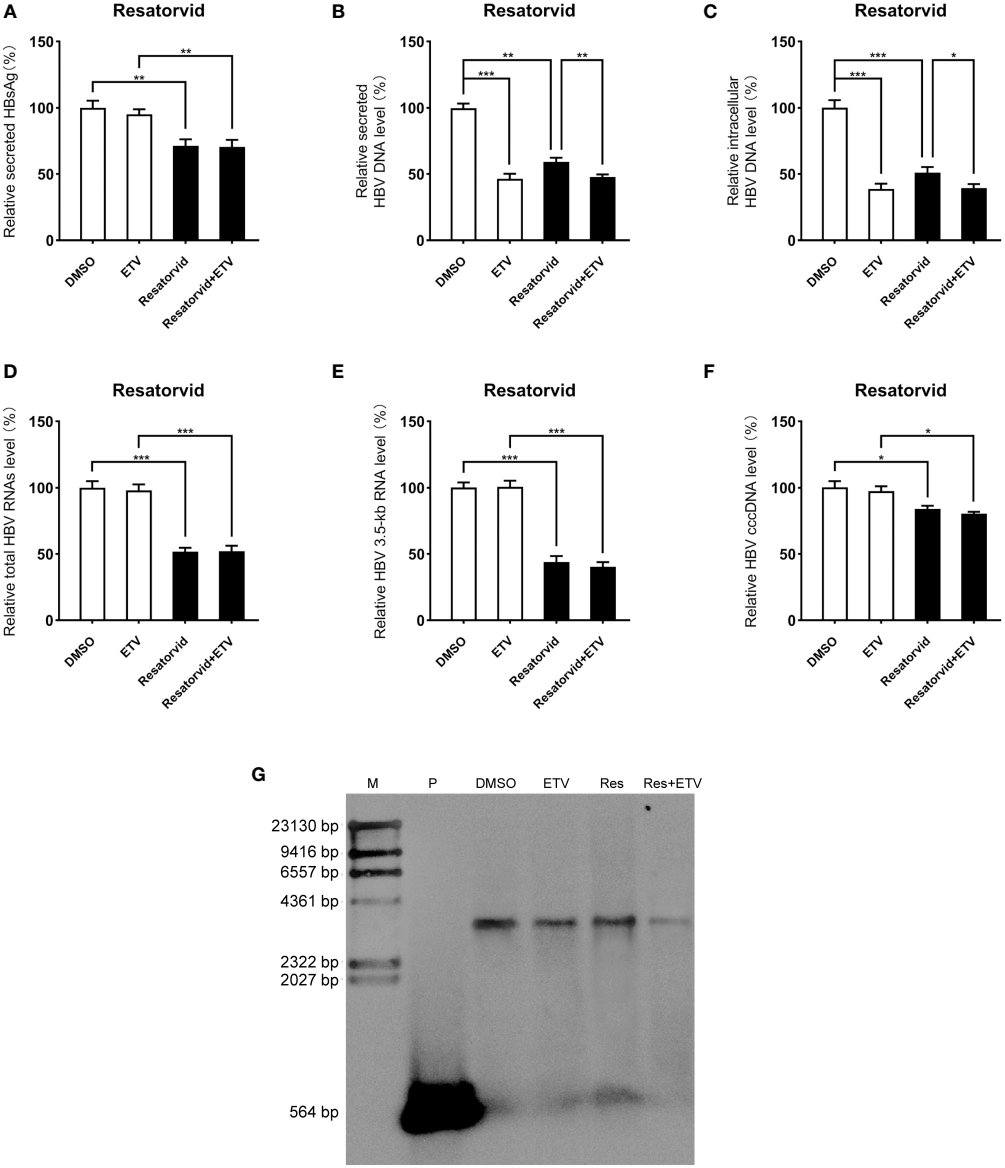
Resatorvid showed anti-HBV activity in the HBV infection model. The HBV-infected cells were treated with 0.1% DMSO, 0.5 nM resatorvid or/and 25 nM ETV for 10 days. **(A)** The results of the ELISA showed that resatorvid decreased the HBsAg level in the supernatant. **(B-E)** Resatorvid significantly inhibited secreted and intracellular HBV DNA, total HBV RNAs, and the 3.5-kb RNA. **(F)** Resatorvid partly decreased the level of cccDNA. **(G)** A Southern blot analysis was performed to determine the reduction of HBV DNA. *, *P* < 0.05; **, *P* < 0.01; ***, *P* < 0.001.

Viral entry into the hepatocyte is mediated by the binding of the NTCP receptor to the pre-S1 domain of L-HBsAg (Ren et al., 2019). Based on the susceptibility of HepG2-NTCP cells to hepatitis B virus particles, a cell model of hepatitis B virus infection was established. To further investigate the effect of plerixafor and resatorvid in the HBV infection model, HepG2-NTCP cells were infected with a normalized amount of HBV particles (1,000 GEq/cell). Then, the HBV-infected HepG2-NTCP cells were treated with 0.5 nM plerixafor/resatorvid and 25 nM ETV as the positive control for 10 days. Plerixafor and resatorvid significantly reduced the levels of HBsAg (*P* < 0.01 and *P* < 0.01) (**Figures 6A**, **7A**). Moreover, the results of real-time PCR analysis showed that the content of HBV DNA in the HepG2-NTCP cells treated with plerixafor and resatorvid decreased significantly (*P* < 0.01 and *P* < 0.01) (**Figures 6B**, **7B**). A similar change in the intracellular HBV DNA content was also found (*P* < 0.001 and *P* < 0.001) (**Figures 6C**, **7C**), which was comparable to the results of the Southern blot assay (**Figures 6G**, **7G**), along with changes in the total HBV RNAs and 3.5-kb RNA (*P* < 0.001 and *P* < 0.001; *P* < 0.001 and *P* < 0.001) (**Figures 6D, E**, **7D, E**). Additionally, the results of the qPCR analysis showed that plerixafor and resatorvid reduced the level of the transcription template cccDNA moderately in the HepG2-NTCP cells (*P* < 0.05 and *P* < 0.05) (**Figures 6F**, **7F**). ETV alone only reduced the level of HBV DNA (*P* < 0.001) without affecting the levels of HBsAg and HBV RNAs, but the combination improved the antiviral activity. Collectively, these results suggested that plerixafor and resatorvid could inhibit HBV replication in the HBV infection model, probably by reducing HBV RNAs and cccDNA levels.

### Plerixafor and resatorvid inhibit HBV replication by targeting EFTUD2

**Figure 8:**
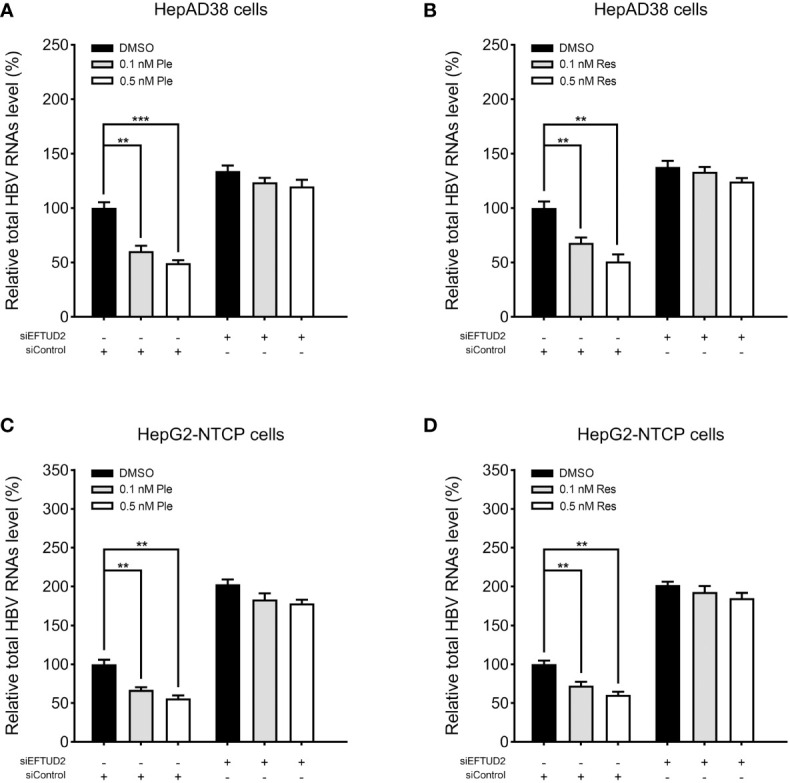
Plerixafor and resatorvid inhibited HBV by targeting EFTUD2. HepAD38 and HepG2-NTCP cells were transfected with siRNAs targeted EFTUD2 (siEFTUD2) or negative control (siControl) , and then, treated with DMSO, plerixafor or resatorvid at indicated concentrations for 7 days. The total HBV RNAs were extracted and quantified by qPCR. The results showed that knocking down EFTUD2 substantially impaired the anti-HBV effect of plerixafor and resatorvid, both in HepAD38 cells **(A, B)** and HepG2-NTCP cells **(C, D)**. **, P < 0.01; ***, P < 0.001.

To determine the mechanism by which plerixafor and resatorvid act on HBV, we silenced EFTUD2 in HepAD38 and HBV-infected HepG2-NTCP cells and tested the antiviral activity of plerixafor and resatorvid. The results showed that knocking down EFTUD2 greatly impaired the effect of plerixafor and resatorvid, both in HepAD38 cells (**Figures 8A, B**) and in HepG2-NTCP cells (**Figures 8C, D**). Taken together, plerixafor and resatorvid act as positive regulators of EFTUD2 and inhibit HBV by upregulating EFTUD2.

## Discussion

Although advancements have been made in the regulation of HBV infection, it is still a threat to public health, especially in less-developed regions. Approximately 292 million people worldwide are living with HBV, and 86 million are affected in China, where only 11% of CHB patients have access to antiviral treatment (Jia et al., 2020). Known treatment strategies mostly involve the use of NAs and interferon-α (IFN-α), which reduce the viral load and improve long-term outcomes, but rarely achieve functional cures (Alonso et al., 2017). Therefore, finding better treatment strategies for HBV infection is extremely important. Although several small molecules have been reported recently, only a few are both safe and efficacious. For example, inarigivir is an agonist of the retinoic acid-inducible gene I (RIG-I) with high anti-HBV efficacy, but it was abandoned because it had adverse effects, and even led to death (Yuen et al., 2019). Vesatolimod (GS-9620) is a safe and well-tolerated TLR-7 agonist and can effectively suppress HBV DNA but does not affect HBsAg (Janssen et al., 2018). The TLR8 agonist selgantolimod (GS-9688) showed seroconversion in the woodchuck model of CHB (Daffis et al., 2021), but limited clinical activity was observed in patients, probably due to dose-limiting events such as gastrointestinal toxicity (Janssen et al., 2021). Thus, searching for new therapeutic targets and agents is necessary. We found a novel class of immunomodulators that targeted EFTUD2 and promoted HBV clearance in different cell models. Furthermore, they showed high anti-HBV efficacy, including the ability to reduce cccDNA.

EFTUD2 is a component of the U5 snRNP, which controls mRNA splicing along with the rest of the spliceosome (Malinová et al., 2017). As a general splicing factor, EFTUD2 participates in various physiological processes such as the organization of myofilaments (Meissner et al., 2009) and P granule development (Updike and Strome, 2009), as well as, pathophysiological processes of some genetic disorders (Löb et al., 2020; Wang et al., 2021; Yang et al., 2022). In this study, we found that silencing EFTUD2 increased the HBV load while overexpressing EFTUD2 reduced the HBV load in different cell models. Moreover, the regulation of EFTUD2 did not significantly affect overall cell viability, suggesting that EFTUD2 can protect against HBV. These results are similar to those regarding the inhibitory effect of EFTUD2 on HCV by modulation of the RIG-I pathway through mRNA splicing (Zhu et al., 2015). Several other viruses have similar characteristics and act on the U5 snRNP components; thus, inducing changes in host cell alternative splicing and affecting virus-host interactions. For example, 3D polymerase from enterovirus 71 directly binds to pre-mRNA processing factor 8 (PRPF8) and disrupts the pre-mRNA splicing processes, contributing to the invasion of the virus (Liu et al., 2014). The NS5 protein of the dengue virus hijacks the splicing machinery by targeting CD2 Cytoplasmic Tail Binding Protein 2 (CD2BP2) and DEAD-Box Helicase 23 (DDX23), creating a favorable environment for the replication of the virus (De Maio et al., 2016). Mammalian orthoreovirus infection leads to a decrease in the expression of EFTUD2 and alterations in cellular splicing, which benefits its oncolytic potential (Boudreault et al., 2022). Several transcriptomic studies have shown that the compatibility between a virus and its host is related to alternative splicing mediated by spliceosomes that are not limited to the U5 snRNP (Batra et al., 2016; Ashraf et al., 2019; Boudreault et al., 2019). We have addressed the role of the U5 core component EFTUD2 in HBV replication in another study (Hu et al., unpublished). In this study, we found that the EFTUD2 gene and protein levels decreased during HBV infection, probably because HBV can downregulate EFTUD2 to reduce its restriction.

A single nucleotide polymorphism analysis showed that the EFTUD2 mutation rs3809756A>C is associated with a decrease in the promoter activity and an increase in the susceptibility to HBV infection (Tian et al., 2022), which supported our findings. In this study, we identified the most active hEFTUD2pro-0.5 kb promoter among the different lengths of the EFTUD2 promoter predicted by bioinformatics, suggesting that it might be a key element in regulating the activity of EFTUD2. Then, the HepG2 cells were stably transfected with a luciferase reporter construct controlled by the hEFTUD2pro-0.5 kb promoter, and the Epro-LUC-HepG2 cell line was constructed to check the promoter activity. By screening 261 compounds from a small-molecule library related to immunity and inflammation, we identified 11 compounds that increased the promoter activity in the Epro-LUC-HepG2 cells by 3–5 folds without showing any signs of obvious cytotoxicity. In these cells, plerixafor and resatorvid showed the most prominent effect on upregulating the expression of EFTUD2.

Plerixafor (AMD3100) is a well-known inhibitor of CXC chemokine receptor 4 (CXCR4) and has specific effects on T4-lymphotropic HIV strains (Hendrix et al., 2000). It was first developed as an anti-HIV drug and has now been repositioned and clinically applied to peripheral blood stem cell mobilization in non-Hodgkin’s lymphoma and multiple myeloma (DiPersio et al., 2009). Plerixafor and its derivatives can also be administered along with other chemicals to increase the effectiveness of treatment in many solid cancers, such as ovarian (Righi et al., 2011), breast (Peng et al., 2015), and pancreatic cancers (Galsky et al., 2014). Resatorvid (TAK-242) is a newly developed, highly selective Toll-like receptor 4 (TLR4) antagonist that is effective for treating pulmonary inflammation (Wang et al., 2016), rheumatoid arthritis (Samarpita et al., 2020), and acute kidney damage (Mohammad et al., 2018). Resatorvid also has hepatoprotective effects against different forms of hepatic dysfunction, such as liver ischemia/reperfusion injury (Shao et al., 2016), along with acute and acute-on-chronic liver failure caused by endotoxemia (Oya et al., 2014; Engelmann et al., 2020). However, the effect of plerixafor or resatorvid on HBV replication in hepatocytes has not been reported. Here, we reported that plerixafor and resatorvid have potent anti-HBV activity *in vitro*.

Our results showed that plerixafor and resatorvid significantly reduced HBsAg secretion even at a low concentration of 0.1 nM with no signs of obvious cytotoxicity in the working concentration range. Furthermore, we found that plerixafor and resatorvid can substantially decrease both intracellular and extracellular HBV DNA levels in a dose-dependent and time-dependent manner. To determine the mechanism, we evaluated the levels of total HBV RNAs and 3.5-kb RNA and found that they decreased similarly. We further investigated the effect of plerixafor and resatorvid on cccDNA, and found a decrease in the cccDNA levels, albeit to a minor degree. However, considering that there was a significant decrease in the ratios of HBV RNAs to cccDNA, the HBV transcription activity was probably suppressed, which explains the decrease in the HBV DNA levels. We speculated the decreased HBV DNA might be due to the diminished upstream HBV RNAs, especially the 3.5-kb RNA.

We proposed that plerixafor and resatorvid act on EFTUD2 rather than other classical targets. Our results showed that silencing EFTUD2 substantially impaired the activity of plerixafor and resatorvid even at a relatively higher concentration of 0.5 nM, but not completely, which might probably be due to the incomplete knockout of EFTUD2. Plerixafor targets CXCR4, but this might not occur in hepatocellular carcinoma (HCC) cells. Many HCC cells express abundant CXCR4 receptors, but its principal ligand C-X-C Motif Chemokine Ligand 12 (CXCL12) is absent, indicating plerixafor inhibits HBV independent of its classical receptor CXCR4 (Li et al., 2014; Kawaguchi et al., 2019). Although some studies reported TLR4 was increased in persistent HBV infection, it was mainly detected in immune cells, not in hepatocytes (Li et al., 2020). In this study, the working concentration of resatorvid was so low that TLR4 was not significantly inhibited (data not shown). What’s more, the synergistic administration of plerixafor and resatorvid for the treatment of HBV was tested, but there was no significant benefit (data not shown). This could be attributed to competition in upregulating EFTUD2.These findings indicated that EFTUD2 is indispensable for the antiviral effect of plerixafor and resatorvid, although the presence of other non-primary targets cannot be excluded.

ETV can act directly on DNA synthesis and strongly inhibit HBV DNA replication. However, it has a minor effect on host immune function or cccDNA micro-chromatin because the transcriptional template cccDNA is found in infected hepatocytes (Marcellin et al., 2008). In this study, we found that the HBV markers in cells and supernatants decreased significantly after treatment with plerixafor or resatorvid alone, but not with ETV alone, which was similar to the findings of previous studies (Marcellin et al., 2008; Buti et al., 2018). Moreover, ETV primarily reduces the HBV DNA levels, while plerixafor and resatorvid primarily decrease HBsAg and HBV RNA levels. Thus, we combined ETV with plerixafor or resatorvid to complement the effects of each other. We found a balanced situation that comprehensively exerted antiviral activity, suggesting that this combination therapy might be better than monotherapy in functional cure.

This study had some limitations besides those mentioned above. First, the effect on the stability of HBV RNAs or the CMV promoter activity in the HepAD38 cells was not further determined, although plerixafor and resatorvid were found to play an anti-HBV role in HepG2-NTCP cells. Also, the antiviral effects of plerixafor and resatorvid on other genotypes or strains, as well as, their effects *in vivo* need to be further studied.

## Conclusion

In this study, we established an EPro-Luc-HepG2 cell line for the first time, which provided a reliable and convenient method for screening small-molecule compounds targeting EFTUD2. The screened small-molecule agents plerixafor and resatorvid significantly upregulated EFTUD2 and decreased HBsAg, HBV DNA, RNAs, and cccDNA levels *in vitro*. The effects of plerixafor and resatorvid complement those of ETV. Thus, these compounds are promising and might be considered while developing complementary or alternative therapeutic strategies for anti-HBV treatment.

## Data availability statement

The original contributions presented in the study are included in the article/**Supplementary Material**. Further inquiries can be directed to the corresponding author.

## Author contributions

JC, YL and CZ conceived the manuscript. JC and YL acquired and analyzed the data, and contributed equally to this work. PH and RX wrote the original draft. HY, WZ, TF, RL and WL revised the manuscript. All authors contributed to the article and approved the submitted version.
