# Supplementary material for: Plerixafor and resatorvid inhibit hepatitis B virus in vitro by upregulating elongation factor Tu GTP-binding domain containing 2
Source: Front Cell Infect Microbiol. 2023 Feb 20;13:1118801. doi: 10.3389/fcimb.2023.1118801 (PMC9986551; doi:10.3389/fcimb.2023.1118801)
Supplement: Supplementary file 1 [file DataSheet_1.docx]

**Table. S1 The primers used in PCR**

| EFTUD2-F | 5’-CAATATCATGGACACTCCAGGAC-3’ |
| --- | --- |
| EFTUD2-R | 5’-CGGTCAATCTTGTTGATGCACA-3’ |
| HBV DNA-F | 5’-CCTAGTAGTCAGTTATGTCAAC-3’ |
| HBV DNA-R | 5’-TCTATAAGCTGGAGGAGTGCGA-3’ |
| Total HBV RNA-F | 5’-ACCGACCTTGAGGCATACTT-3’ |
| Total HBV RNA-R | 5’-GCCTACAGCCTCCTAGTACA-3’ |
| HBV 3.5-kb RNA-F | 5’-GCCTTAGAGTCTCCTGAGCA-3’ |
| HBV 3.5-kb RNA-R | 5’-GAGGGAGTTCTTCTTCTAGG-3’ |
| HBV cccDNA-F | 5’-CTCCCCGTCTGTGCCTTCT-3’ |
| HBV cccDNA-R | 5’-CCCCAAAGCCACCCAAG-3’ |
| HBV cccDNA probe | 5’-TTCATCCTGCTGCTATGCCTGATCTTCTTG-3’ |
| GAPDH-F | 5’-ACAGTCCATGCCATCACTGCC-3’ |
| GAPDH-R | 5’-GCCTGCTTCACCACCTTCTTG-3’ |

**Fig. S1** **Identification of the EFTUD2 promoters and the LV6-Epro0.5-LUC luciferase reporter plasmid**


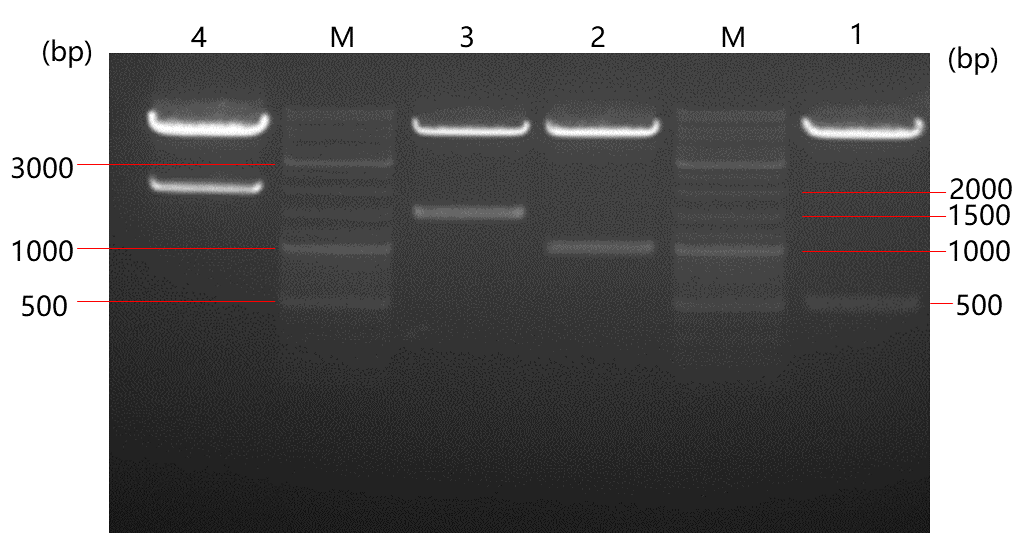
S1 A


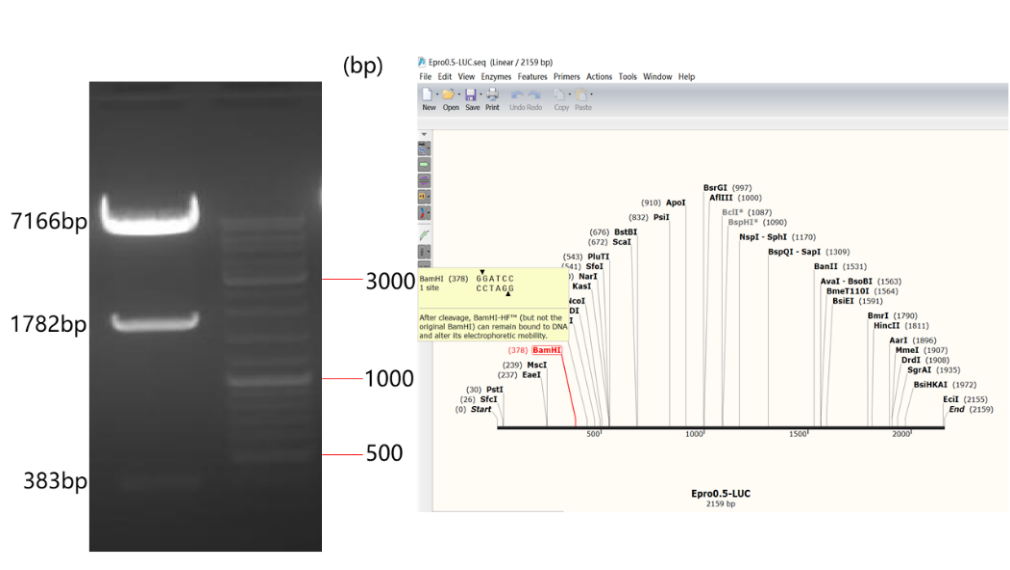
S1 B

**Fig. S1 Identification of the EFTUD2 promoters and the LV6-Epro0.5-LUC luciferase reporter plasmid.** (A) The EFTUD2 promoter-luciferase constructor was verified by performing agarose gel electrophoresis. (B) The hEFTUD2pro-0.5 kb promoter-luciferase plasmid was verified by performing agarose gel electrophoresis.

**Fig. S2 The cytotoxicity of the screened compounds in Epro-LUC-HepG2 cells (partly shown).**


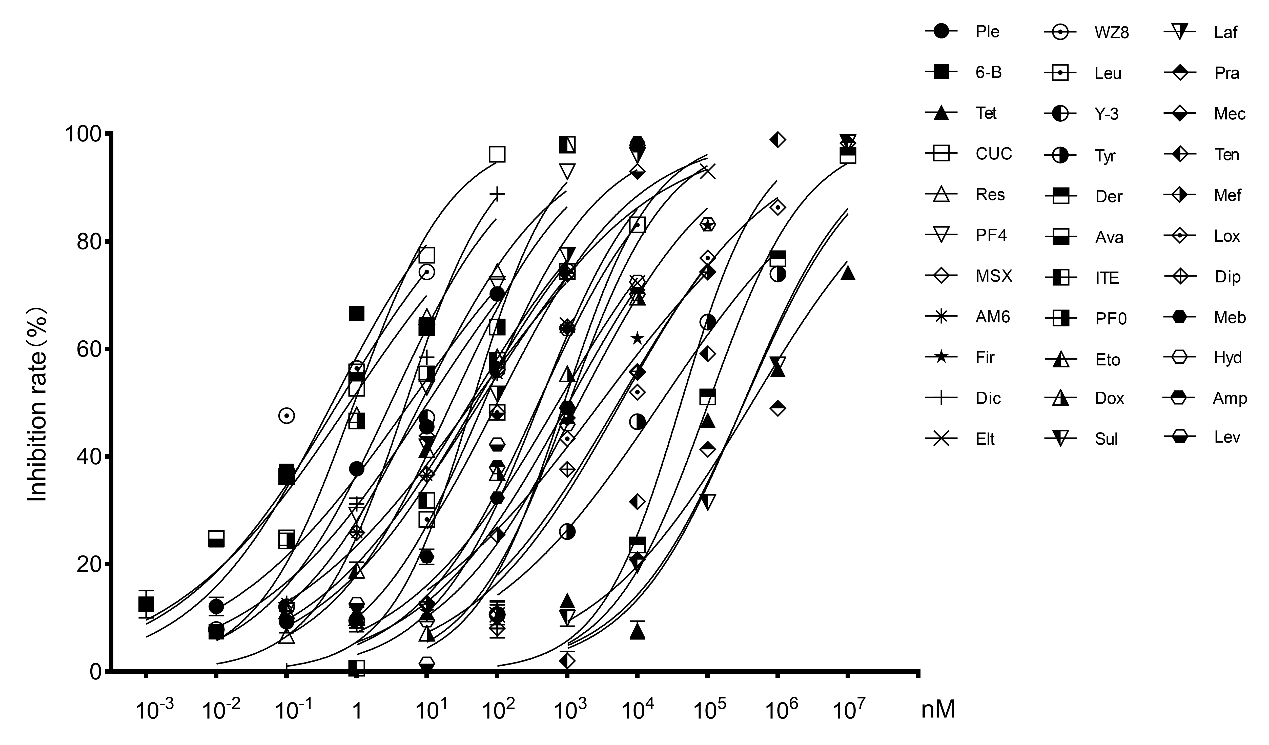


S2

**Fig. S2 The cytotoxicity of the screened compounds in Epro-LUC-HepG2 cells (partly shown).** Epro-LUC-HepG2 cells were treated with The cytotoxicity of these compounds was evaluated by performing the MTT assay in Epro-LUC-HepG2 cells.

**Fig. S3 The characteristics of plerixafor and resatorvid.**

S3 A


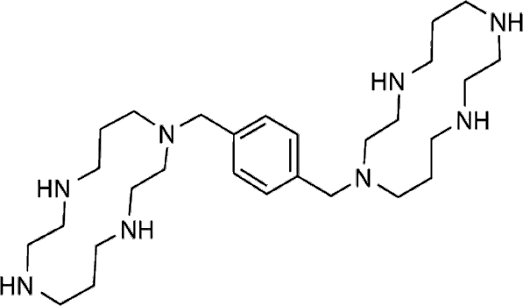

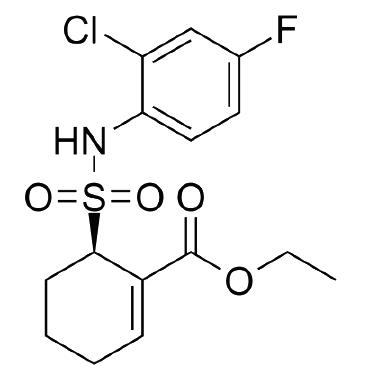


S3 B

**Plerixafor**

Formular Weight = 502.78

Cas No.: 110078-46-1

**Resatorvid**

Formular Weight = 361.82

Cas No.: 243984-11-4

S3 D

S3 C


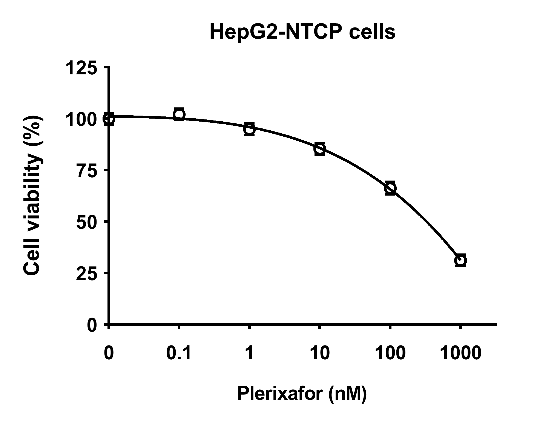

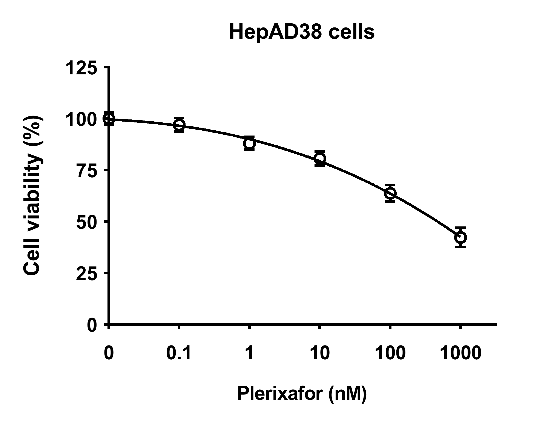


S3 F

S3 E


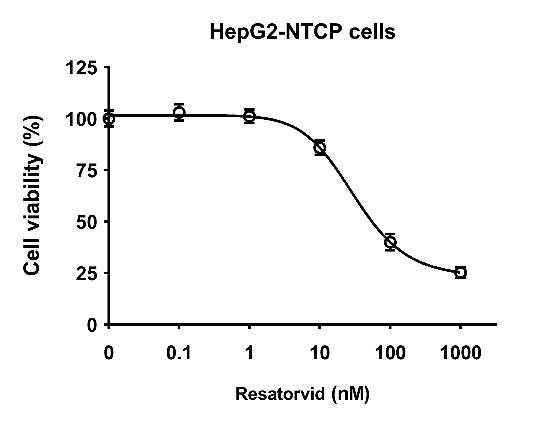

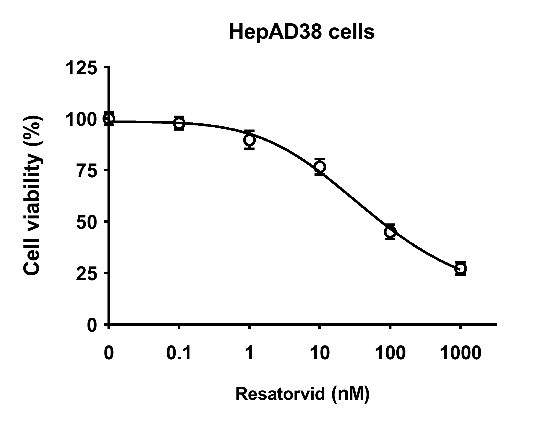


**Fig. S3 The characteristics of plerixafor and resatorvid.** (A-B) The structural formula and basic information of plerixafor and resatorvid. (C-F) The HepAD38 cells and HepG2-NTCP cells were treated with the indicated concentrations of plerixafor and resatorvid for 3 days. The effects of plerixafor and resatorvid on cell viability were determined by performing the MTT assay.
